# Impact of Patient Education on Quality of Life in Lung Cancer: A Narrative Review of Studies Using QLQ-C30 and QLQ-LC13

**DOI:** 10.7759/cureus.108686

**Published:** 2026-05-11

**Authors:** Anastasios N Moysiadis, Konstantinos Kontzoglou, Dimitrios Dimitroulis, Sofoklis Mitsos, Nikolaos L Korodimos, Periklis Tomos

**Affiliations:** 1 Department of Surgery, General University Hospital Attikon, Athens, GRC; 2 School of Medicine, National and Kapodistrian University of Athens, Athens, GRC; 3 2nd Department of Propaedeutic Surgery, Laiko Hospital, Athens Medical School, National and Kapodistrian University of Athens, Athens, GRC; 4 Department of Thoracic Surgery, General University Hospital Attikon, Athens, GRC

**Keywords:** lung cancer, patient education, postoperative recovery, psychosocial support, qlq-c30, qlq-lc13, quality of life, rehabilitation, supportive care, treatment recovery

## Abstract

This narrative review evaluates the impact of patient education and supportive interventions on the quality of life of patients with lung cancer, focusing on studies utilizing the European Organisation for Research and Treatment of Cancer (EORTC) QLQ-C30 and QLQ-LC13 instruments. A structured literature search was conducted across major databases, and studies assessing educational, physical, and psychosocial interventions were included. Findings suggest that patient education is associated with improvements in symptom management, emotional well-being, and overall quality of life, while exercise and psychosocial support contribute to enhanced physical functioning and reduced symptom burden. Despite heterogeneity among studies, the overall evidence indicates that integrating structured educational strategies into routine care may improve patient-reported outcomes, quality of life, and functional adaptation across diverse stages of lung cancer care, including surgical, systemic, and supportive treatment settings.

## Introduction and background

Lung cancer continues to represent a major global health challenge and is a leading cause of cancer-related death worldwide. Beyond its high mortality, the disease is associated with a substantial burden on patients’ quality of life (QoL) due to both disease-related symptoms and treatment-related side effects [1,2].

Despite growing evidence, the overall impact of patient education on QoL in lung cancer remains variably reported. This narrative review aims to synthesize evidence on the impact of educational and supportive interventions on QoL in lung cancer patients using validated European Organisation for Research and Treatment of Cancer (EORTC) instruments.

Several factors may impact the QoL of patients with lung cancer across the continuum of care, including surgical recovery, systemic treatment, symptom burden, and psychosocial adaptation. Recovery is affected by both the type of surgery and personal characteristics such as age, fitness level, comorbidities, nutrition, and the patient's mental condition [2,3].

Acceptance of the condition constitutes a key psychological factor in the healing process. Greater awareness and acceptance are associated with improved adherence to therapy, reduced anxiety, enhanced emotional well-being, and overall better QoL, as shown by several studies [4]. According to the Illness Cognition Theory, perceiving an illness as controllable and comprehensible fosters self-care behaviors and psychological adaptation [5]. Patient education can support acceptance by reducing uncertainty and concern, while providing information about the diagnosis, treatment, and procedures involved [6,7].

QoL has become a central outcome in oncology, reflecting not only survival but also patients’ physical, emotional, cognitive, and social functioning. Standardized instruments such as the EORTC QLQ-C30 and its lung cancer-specific module, QLQ-LC13, are widely used to assess these multidimensional aspects [8]. Research using these scales for patient education has shown that information and guidance enhance functional scale scores and mitigate symptoms, facilitating swifter postoperative recovery and reducing the need for readmissions [8,9]. Importantly, the educational and supportive needs of lung cancer patients vary according to treatment modality, with distinct requirements in surgical and systemic therapy settings.

Pain perception is another crucial part of rehabilitation after surgery, as it affects patients’ physical activities, breathing, and mental condition. Regular assessment using pain scales and QLQ-C30/LC13 scores facilitates individualized pain treatment and improves overall QoL [10].

In recent years, increasing attention has been given to supportive care interventions, particularly patient education. For the purposes of this review, patient education refers to structured interventions designed to improve patients’ understanding of disease, treatment, symptom management, self-management skills, and treatment adherence through informational, behavioral, or psychosocial support strategies [5-7,11,12]. Such interventions aim to enhance patient engagement, self-efficacy, and adherence to treatment, thereby improving health-related outcomes [13].

In addition, exercise-based interventions and psychosocial support have been shown to positively influence QoL outcomes [14,15]. This review considers patient education and supportive interventions across the entire lung cancer care continuum, including surgical, systemic therapy, and advanced disease settings, to provide a comprehensive synthesis of their impact on QoL. Importantly, QoL determinants differ across treatment settings, with surgical patients primarily experiencing recovery-related functional limitations, while patients receiving systemic therapy are more affected by cumulative treatment-related toxicity and symptom burden. This distinction is essential for interpreting QoL outcomes across studies included in this review. Supportive care and educational needs may differ substantially between patients undergoing surgical treatment and those receiving systemic therapies for advanced disease. Therefore, individualized supportive strategies across different treatment settings are essential to optimize health-related QoL throughout the lung cancer care continuum.

The QLQ-C30 and QLQ-LC13 questionnaires

QoL has become a central outcome in lung cancer care, reflecting physical, emotional, cognitive, and social well-being. The EORTC QLQ-C30 is a validated cancer-specific questionnaire assessing global health status, functional domains, and symptom burden, while the lung cancer-specific QLQ-LC13 module evaluates symptoms including dyspnea, coughing, chest pain, and treatment-related side effects. These instruments are widely used across different stages of lung cancer care to assess multidimensional QoL outcomes and provide standardized measures for evaluating the effectiveness of educational and supportive interventions in lung cancer patients [8,16-19]. These instruments are particularly valuable in lung cancer research as they capture clinically meaningful changes across different treatment settings, including surgical recovery and systemic therapy.

## Review

Methodology

Study Design

**Figure 1:**
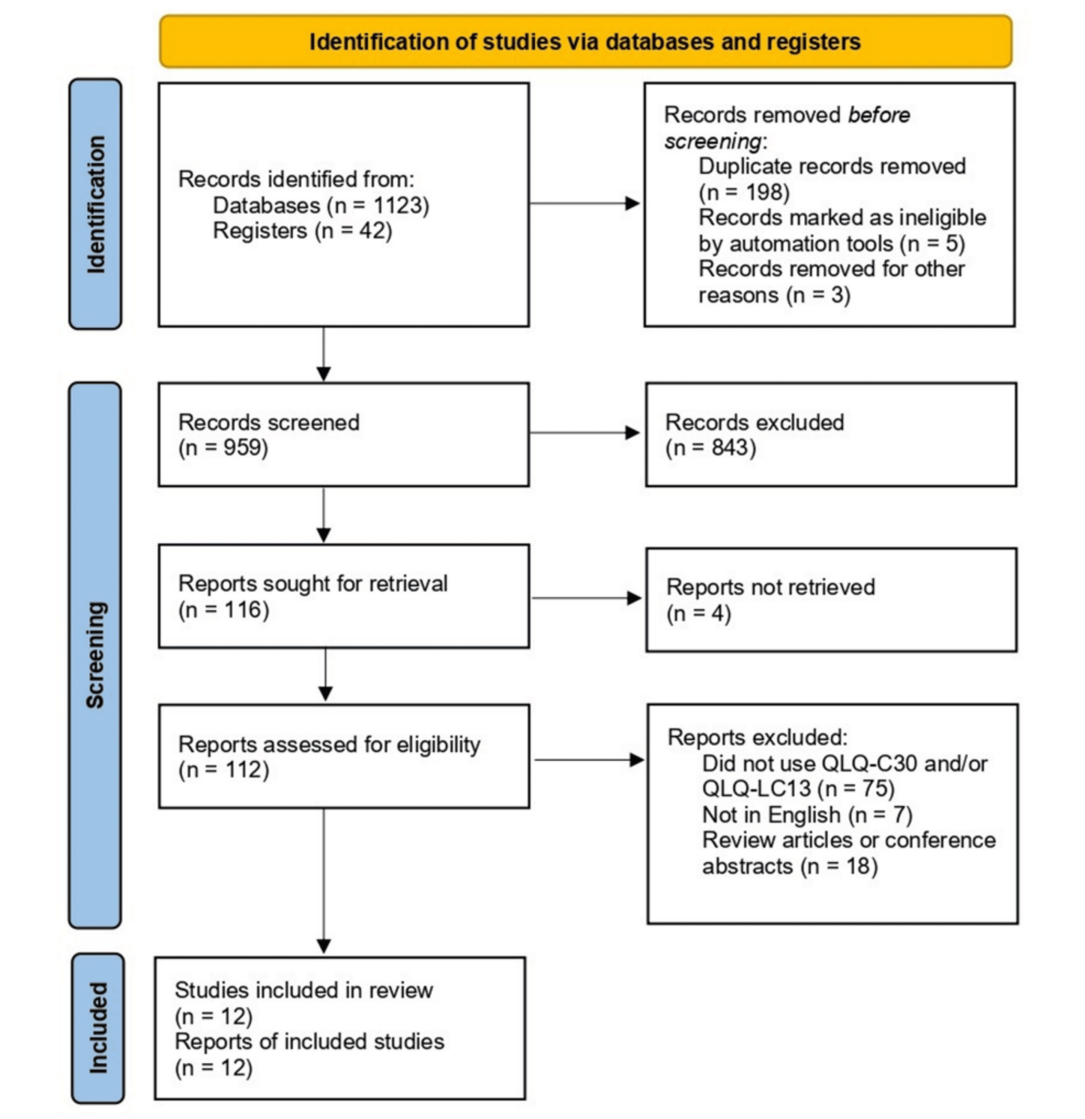
PRISMA 2020 flow diagram of the study selection process. PRISMA: Preferred Reporting Items for Systematic Reviews and Meta-Analyses

This narrative review was conducted using a structured literature search strategy in accordance with the Preferred Reporting Items for Systematic Reviews and Meta-Analyses (PRISMA) 2020 guidelines. The study selection process is illustrated in Figure 1 [20]. Due to heterogeneity in study design, interventions, and outcome reporting, quantitative synthesis was not feasible.

Eligibility criteria included studies involving adult patients with lung cancer that evaluated structured educational, psychosocial, physical rehabilitation, or other supportive care interventions, and reported QoL outcomes using validated instruments (EORTC QLQ-C30 and/or QLQ-LC13). No restrictions were applied regarding disease stage or treatment modality, provided that studies aligned with the predefined scope of supportive interventions across the lung cancer care continuum. For the purposes of this review, supportive care was defined as interventions aimed at improving symptom management, self-care capacity, functional adaptation, psychosocial well-being, or treatment adherence throughout the disease trajectory.

Search Strategy

A comprehensive literature search was performed in electronic databases, including PubMed, Scopus, and Web of Science. The search period covered studies published from 1993 to 2025. Search terms included “lung cancer”, “quality of life”, “QLQ-C30”, “QLQ-LC13”, “patient education”, “supportive care”, “rehabilitation”, “psychosocial support”, and “treatment recovery”. Boolean operators (AND, OR) were used to refine the search. A broad search strategy was intentionally used to capture educational and supportive interventions across multiple lung cancer treatment settings, including surgical, systemic, rehabilitative, and survivorship care.

Inclusion and Exclusion Criteria

The studies were selected based on the following criteria: (a) adult patients with lung cancer, (b) use of the EORTC QLQ-C30 and/or QLQ-LC13 questionnaires, and (c) evaluation of structured interventions including educational, psychosocial, physical rehabilitation, or supportive care approaches aimed at improving QoL across any stage of lung cancer care, including surgical, systemic, and survivorship settings.

No restrictions were applied regarding disease stage or treatment modality, provided that studies evaluated patient education or supportive interventions in lung cancer populations across any phase of the disease continuum. Studies not meeting these criteria, non-English publications, and those lacking relevant outcome data were excluded, as well as reviews and conference abstracts.

Study Selection

Two independent reviewers screened titles and abstracts. Full texts were assessed for eligibility. Disagreements were resolved through discussion or by a third reviewer.

Data Extraction

Data extracted included study design, sample size, intervention type, and QoL outcomes. Emphasis was placed on functional scales, symptom burden, and educational impact. Quantitative QoL outcomes derived from validated EORTC instruments, including reported domain trends and score changes where available, were extracted for comparative synthesis.

Outcome Measures

QoL outcomes were primarily assessed using the EORTC Quality of Life Questionnaire Core 30 (EORTC QLQ-C30) and the lung cancer-specific module QLQ-LC13. The EORTC QLQ-C30 is a standardized instrument for measuring QoL in oncology, comprising 30 items grouped into three scales: functional, symptom, and global health status. The functional scales assess physical, emotional, cognitive, and social functioning. The symptom scales capture treatment- and disease-related symptoms such as pain, fatigue, nausea, and vomiting while the global health status scale provides an overall measure of patients’ perceived QoL [8]. The QLQ-LC13 is a supplementary lung cancer-specific module designed to evaluate symptoms directly related to lung cancer and its treatment, including cough, hemoptysis, dyspnea, chest pain, and analgesic use [16]. The use of QLQ-C30 and QLQ-LC13 has demonstrated effectiveness in assessing the QoL among lung cancer patients. Their application in patient education studies enables the systematic evaluation of intervention outcomes, the monitoring of postoperative recovery, and the facilitation of evidence-based clinical practice.

Furthermore, the widespread use of these instruments ensures accurate and reliable measurement, which cuts down on discrepancies that come from cultural or language issues [8,16,17]. In studies focusing on patient education levels, the QLQ-C30 and QLQ-LC13 have provided valuable data regarding the impact of educational programs on QoL. Simultaneously, enhancement in QoL indicators correlates with diminished postoperative readmissions, improved treatment adherence, and favorable modifications in clinical indicators, including respiratory function and standard biometric measures [17-19].

Quality Assessment and Risk of Bias

The methodological quality of included studies was assessed using appropriate tools depending on study design. Randomized controlled trials were evaluated using the Cochrane Risk of Bias Tool, while observational studies were assessed using Risk Of Bias In Non-randomized Studies of Interventions (ROBINS-I). Studies were categorized as low, moderate, or high risk of bias. Assessment was performed independently by two reviewers.

Data Synthesis

Due to heterogeneity in study design, interventions, and outcome measures, a meta-analysis was not performed. Instead, findings were synthesized narratively. The findings were grouped into themes: educational interventions, physical rehabilitation, and psychosocial outcomes.

Results

*Educational Interventions and Symptom Managemen*t

A total of 12 studies met the inclusion criteria and were included in the final synthesis. The included studies exhibited substantial heterogeneity in sample sizes, ranging from small pilot trials to large multicenter randomized controlled studies, with a combined population of 2,642 patients with lung cancer. Studies were conducted between 2016 and 2025, with variability observed in intervention types, duration, and outcome reporting. The included studies covered a broad range of clinical settings, including perioperative care, systemic therapy, rehabilitation, and advanced disease populations.

Across studies, educational and supportive interventions were generally associated with improvements in multiple domains of the EORTC QLQ-C30 and QLQ-LC13, including emotional functioning, symptom burden, dyspnea, fatigue, and global health status. Reductions in lung cancer-specific symptoms such as cough, chest pain, and dyspnea were also reported in studies using the QLQ-LC13 module. Clinically meaningful differences in QLQ-C30 scores are commonly considered to approximate 10 points; however, this threshold could not be consistently applied due to heterogeneity across studies and variability in statistical reporting.

Educational interventions were associated with improved patient knowledge and self-management capacity, while supportive interventions were associated with better symptom control and functional outcomes. These effects were most consistently observed in emotional functioning, symptom burden, and global health status domains.

Pain Management and Functional Recovery

Pain is a significant and persistent symptom in patients undergoing lung cancer surgery and postoperative recovery, substantially affecting mobility, respiratory function, and overall QoL. Pain management is crucial not only for pain relief but also for improving mobility, respiratory function, and overall QoL [7]. Pain evaluations are often performed with pain scales, such as the Visual Analogue Scale (VAS) or the Numerical Rating Scale (NRS), which provide quantitative data on pain intensity, hence enabling correlation with the results of the QLQ-C30 and LC13 scales. Preoperative education of patients has been shown to significantly influence their emotional response and pain management. Evidence indicates that patients who participate in educational programs report lower pain scores on the QLQ-C30 and QLQ-LC13, reduced reliance on analgesics, and improved overall functioning [7,21].

Patient education should address the expected progression of pain, respiratory procedures, and ways to prevent complications such as pulmonary infections, while also emphasizing self-management strategies. These strategies may include relaxation methods, proper body alignment, and gentle mobility exercises.

There are three primary domains of patient education. The first is cognitive education about postoperative pain and the importance of following pain management protocols. The second is behavioral education, which focuses on practical strategies to improve comfort and mobility, such as progressive exercise, breathing techniques, and relaxation practices. The third is emotional support, which addresses pain-related stress and anxiety through interventions such as mindfulness, relaxation training, and psychotherapy. Studies have shown that emotional support can reduce pain perception and enhance illness acceptance, as reflected in improved functional scores on the QLQ-C30/LC13 [14].

Clinical and Biometric Outcomes

Clinical and biometric outcomes differed across treatment settings. In postoperative populations, educational interventions mainly focused on respiratory recovery, mobility, and prevention of complications, whereas in advanced disease settings, emphasis was placed on symptom monitoring, treatment adherence, nutritional status, and maintenance of functional capacity during systemic therapy. Educational interventions have a significant influence on both biometric and clinical indicators, as well as on patients’ overall QoL. Through structured health education programs, patients are encouraged to monitor and regulate key biometric parameters such as body weight, body mass index (BMI), dietary habits, and levels of physical activity. Continuous monitoring of these indicators not only promotes healthier lifestyle choices but also supports the early detection of deviations that may signal emerging health risks. This proactive approach aligns with established clinical guidelines aimed at maintaining physiological stability and preventing disease progression [4].

Furthermore, educational programs have a measurable impact on QoL by enhancing physical, social, and emotional functioning. Patients who actively engage in educational activities often report reduced symptom burden, including less dyspnea and pain, as well as improved coping mechanisms and psychological well-being. This suggests that education not only supports physiological health but also contributes to emotional resilience and social adaptability [22,23].

From a clinical perspective, education facilitates better understanding and management of respiratory function, regular participation in laboratory testing, and early identification of potential complications. Educated patients are more likely to comply with recommended diagnostic and preventive measures, leading to improved clinical outcomes and reduced morbidity [24].

In addition, specific educational strategies such as training in breathing techniques, recognition of symptom patterns, and adherence to prescribed medications play a critical role in disease management. These strategies empower patients to take an active role in their care, enabling more effective self-regulation and promoting treatment adherence. Consequently, education becomes a vital component of comprehensive healthcare, fostering both clinical improvement and enhanced patient autonomy [14].

The correlation of biometric and clinical indicators with QoL is assessed using the QLQ-C30/LC13. Patients who adhere to educational guidelines show improvements in physical functioning, reduced symptoms such as dyspnea and pain, and higher scores in social and emotional functioning [25]. In addition, continuous monitoring enables the personalization of interventions and the timely identification and management of complications, thereby enhancing the effectiveness of educational programs.

Psychosocial and supportive outcomes

Postoperative and Rehabilitation-Based Support

Lung cancer is one of the major causes of cancer-related morbidity and mortality in the world, with significant physical, psychological, and social consequences on the patient [22]. There has been improvement in the survival rate, due to improved surgical, chemotherapeutic, and immunotherapeutic interventions; however, survival is frequently accompanied by postoperative and treatment-related physical and psychosocial burden [26,27]. Psychosocial support, patient education, and organized rehabilitation programs have become key measures in reducing these effects [23,28]. Education improves disease and treatment knowledge, enhances self-care management, reduces complications, and allows the patient to become an active member in the recovery process [29,30]. It has been indicated that better-informed patients demonstrate higher levels of disease acceptance, reduced anxiety, and improved compliance with therapeutic regimens [29].

Psychosocial Support During Systemic Therapy

According to research, psychosocial and supportive interventions were associated with improvements in emotional well-being, reduced anxiety levels, and enhanced social functioning and symptom monitoring [28]. The literature emphasized the effectiveness of the stepped-care psychological programs, as they provided interventions based on the levels of distress, are patient-reported, and thus maximized the effectiveness of the use of resources, as well as enhanced emotional well-being.

Psychosocial support is increasingly being delivered through digital and remote care platforms. Jing et al. employed electronic patient-reported outcomes to inform the management of symptoms, which proved to increase the QoL and decrease the number of complications associated with treatment [31]. These outcomes were consistently reflected across multiple QLQ-C30 domains, highlighting the importance of structured psychosocial support and symptom monitoring throughout the lung cancer care continuum.

Physical Rehabilitation and Exercise-Based Interventions

Several studies assessing exercise-based interventions in postoperative and systemic therapy lung cancer populations demonstrated improvements in physical functioning and reductions in fatigue. Exercise interventions were also associated with better maintenance of functional capacity over time in postoperative lung cancer patients [22,23]. Messaggi-Sartor et al. reported a pilot randomized clinical trial where the combination of aerobic and high-intensity respiratory muscle training decreased the burden of symptoms and improved patient resilience and self-management [22]. Granger et al., in a postoperative lung cancer population, have shown that home-based educational and self-management interventions after the resection of the lungs had a significant positive effect on patient-reported outcomes, such as functional capacity and psychological well-being [27]. Jonsson et al. and Bloch et al. reported that consistent physical activity in postoperative lung cancer patients, continued at three and 12 months, was associated with increased QoL scores, especially in physical and social functioning [23,32]. Exercise-based interventions were not limited to postoperative recovery settings but have also been evaluated in patients with advanced or metastatic non-small-cell lung cancer (NSCLC) receiving systemic therapies, although the magnitude and clinical context of benefit may differ across disease stages.

Interventions in Advanced Disease Settings

Patients with advanced or metastatic NSCLC receiving systemic therapy or immunotherapy also report QoL benefits from structured exercise programs, particularly in maintaining functional capacity during treatment [30,33,34]. Taken collectively, these results support the significance of exercise as a part of a holistic treatment approach, as it supports both physiological and psychosocial adaptation.

Differences in QoL outcomes were also observed according to treatment modality and disease stage, with postoperative surgical populations demonstrating more consistent improvements in physical recovery outcomes, whereas patients receiving systemic therapy showed more variable but still clinically relevant functional maintenance, further supporting the need for individualized supportive care strategies.

Multimodal and Integrated Supportive Care Approaches

Multicomponent (education, physical activity, and psychosocial support) comprehensive rehabilitative approaches have shown synergies in improving QoL. The results of Granger et al. demonstrated that home-based exercise and self-management education reduced postoperative fatigue, increased physical activity compliance, and improved emotional well-being [27]. Fiteni et al. highlighted that multimodal approaches may be particularly beneficial in elderly patients, as they may alleviate physical weakness and improve psychological resilience [35]. Gu et al. demonstrated the potential benefits of integrative approaches and reported better postoperative recovery and QoL among patients of NSCLC with the application of Chinese medicine, in addition to conventional treatment [36]. The research findings indicate that holistic care, which helps to fulfill all the requirements of patients: physical, emotional, and educational needs, is the best way to maximize patient results and promote long-term recovery [37].

Patient education, guided physical activity, and psychosocial interventions collectively contribute to improved disease acceptance and QoL in patients with lung cancer. Randomized trials, cohort studies, and pilot interventions have shown that well-informed and proactive patients have lower symptom burden, better emotional resilience, and better functional recovery [22,23,26-28,30,32,34-37].

Table 1 summarizes study characteristics and key QoL domains affected by educational and supportive interventions.

**Table 1:** Characteristics of included studies. Where detailed numerical reporting was unavailable, outcome domains are summarized narratively based on reported QLQ-C30/QLQ-LC13 trends.

Author (year)	Country	Sample size (N)	Study design	Intervention/main focus	Reported quality of life outcomes (QLQ-C30/QLQ-LC13 trends)
Cetkin and Tuna (2019) [10]	Turkey	60	Quasi-experimental study	Preoperative patient education	Reduced anxiety and improved respiratory outcomes, leading to better global quality of life
Messaggi-Sartor et al. (2019) [22]	Spain	37	Pilot randomized clinical trial	Post-surgical exercise	Improved physical functioning, reduced fatigue, enhanced symptom control
Jonsson et al. (2025) [23]	Sweden	83	Cross-sectional study	Physical activity and quality of life	Higher physical activity associated with improved physical/social functioning
Granger et al. (2024) [27]	Australia	116	Randomized clinical trial	Home-based rehabilitation	Improved recovery, reduced fatigue, enhanced functional capacity and quality of life
Takano et al. (2021) [28]	Japan	200	Randomized controlled trial	Self-help workbook	Improved emotional well-being, symptom monitoring, maintained quality of life
Waterhouse et al. (2024) [30]	International	345	Multicenter randomized controlled trial	Targeted therapy	Maintained or improved quality of life during systemic treatment
Jing et al. (2025) [31]	China	355	Randomized controlled trial	ePRO monitoring	Better symptom control, improved patient-reported well-being
Bloch et al. (2025) [32]	Denmark	218	Secondary analysis	Exercise in advanced cancer	Reduced pain and slowed deterioration in quality of life
Makharadze et al. (2023) [34]	Multinational	466	Phase III multicenter randomized controlled trial	Immunotherapy and chemotherapy	Improved survival with maintained quality of life despite advanced treatment burden
Fiteni et al. (2016) [35]	France	451	Randomized controlled trial	Chemotherapy regimens	Different quality of life profiles observed depending on treatment profile
Gu et al. (2025) [36]	China	99	Randomized controlled trial	Integrative rehabilitation	Improved postoperative functioning and symptom relief
Kenis et al. (2025) [37]	Belgium	212	Multicenter randomized controlled trial	Geriatric assessment	Improved quality of life in eldery populations

Risk of Bias Assessment

The methodological quality of the included studies was assessed using appropriate tools based on study design. Most randomized controlled trials were judged to have a low risk of bias, particularly multicenter and phase III trials. However, some smaller or pilot randomized studies demonstrated moderate risk due to potential limitations in blinding or sample size. Observational and non-randomized studies were generally associated with a moderate risk of bias, mainly due to confounding factors and study design limitations. A detailed summary of the risk of bias assessment is presented in Table 2.

**Table 2:** Risk of bias assessment of included studies. ROBINS-I: Risk Of Bias In Non-randomized Studies of Interventions

Study	Design	Assessment tool	Overall risk of bias
Cetkin and Tuna (2019) [10]	Quasi-experimental study	ROBINS-I	Moderate
Messaggi-Sartor et al. (2019) [22]	Pilot randomized controlled trial	Cochrane Risk of Bias Tool	Moderate
Jonsson et al. (2025) [23]	Cross-sectional study	ROBINS-I	Moderate
Granger et al. (2024) [27]	Randomized controlled trial	Cochrane Risk of Bias Tool	Low
Takano et al. (2021) [28]	Randomized controlled trial	Cochrane Risk of Bias Tool	Low
Waterhouse et al. (2024) [30]	Multicenter randomized controlled trial	Cochrane Risk of Bias Tool	Low
Jing et al. (2025) [31]	Randomized controlled trial	Cochrane Risk of Bias Tool	Low
Bloch et al. (2025) [32]	Secondary analysis	ROBINS-I	Moderate
Makharadze et al. (2023) [34]	Phase III multicenter randomized controlled trial	Cochrane Risk of Bias Tool	Low
Fiteni et al. (2016) [35]	Randomized controlled trial	Cochrane Risk of Bias Tool	Moderate
Gu et al. (2025) [36]	Randomized controlled trial	Cochrane Risk of Bias Tool	Low
Kenis et al. (2025) [37]	Multicenter randomized controlled trial	Cochrane Risk of Bias Tool	Low

Clinical Implications and Future Directions

A combination of systematic patient education and systematic QoL assessment provides a holistic and patient-centered approach to lung cancer. Incorporating a combination of psychological adaptation, the understanding of the disease, and coping with the symptoms, healthcare providers will be able to enhance the outcomes of postoperative care and overall well-being. The QLQ-C30 and QLQ-LC13 questionnaires are used not only to report patients’ outcomes but also to determine the impact of clinical intervention in modern oncological care. Further studies should focus on tailor-made educational interventions and technology-facilitated models to improve the comprehensive approach to lung cancer patients. Future prospective studies incorporating standardized QLQ-C30 and QLQ-LC13 assessments may provide more robust comparative quantitative evidence.

Discussion

This review synthesizes current evidence regarding the impact of patient education and supportive interventions on QoL in patients with lung cancer. Findings were broadly observed across educational, physical rehabilitation, psychosocial, and multimodal supportive care domains. The included studies encompassed heterogeneous populations across different disease stages and treatment modalities, which enhances the generalizability of findings but limits direct comparability across studies.

Overall, the available evidence suggests that these interventions are associated with improvements across multiple domains of health-related QoL. Although quantitative synthesis was constrained by inconsistent reporting, most studies demonstrated favorable trends in validated EORTC QLQ-C30 and QLQ-LC13 domains, supporting the clinical relevance of educational and supportive approaches [8,16].

Patient education appears to play a key role by improving disease understanding and facilitating active patient participation in care. Cetkin and Tuna [10] demonstrated that preoperative educational interventions significantly reduced anxiety and improved respiratory outcomes, contributing to better overall QoL. Similarly, Takano et al. [28] reported that structured self-help educational interventions during chemotherapy improved emotional functioning and helped maintain QoL, while Jing et al. [31] showed that electronic patient-reported outcome monitoring enhanced symptom control and psychological well-being. Collectively, these findings suggest that improved knowledge and structured guidance support better self-management and treatment adherence [12,13].

Exercise-based interventions were consistently associated with improvements in physical functioning and reductions in fatigue, both of which are key determinants of overall QoL in lung cancer patients. Messaggi-Sartor et al. [22] demonstrated that combined aerobic and respiratory muscle training improved functional capacity and reduced symptom burden following surgery. Similarly, Granger et al. [27] reported that home-based rehabilitation significantly improved recovery and reduced fatigue after lung cancer resection. Jonsson et al. [23] found that higher levels of physical activity were associated with better physical and social functioning, while Bloch et al. [32] showed that exercise slowed the deterioration of QoL in patients with advanced disease. These findings collectively highlight the importance of structured physical activity across different disease stages.

Psychosocial interventions also contributed to improved emotional and social functioning. Cetkin and Tuna [10] reported reductions in anxiety following preoperative education, while Takano et al. [28] demonstrated improvements in emotional well-being during chemotherapy through structured self-management support. Furthermore, Jing et al. [31] highlighted that ePRO-based interventions provided real-time symptom monitoring and psychological support, leading to better patient-reported outcomes. These results emphasize the importance of addressing psychological distress as part of comprehensive lung cancer care.

These findings are further supported by broader systematic reviews and meta-analytic evidence demonstrating that psychosocial and supportive care interventions improve emotional well-being, distress management, and health-related QoL across oncology populations, including lung cancer care settings [38,39]. Differences in QoL outcomes were also observed according to treatment modality and disease stage, further supporting the need for individualized supportive care strategies. Specifically, postoperative surgical populations demonstrated more consistent improvements in physical recovery outcomes following exercise-based interventions, whereas patients receiving systemic therapy or immunotherapy exhibited more heterogeneous but clinically meaningful benefits, primarily related to functional maintenance, symptom burden, and treatment tolerance.

In advanced disease settings, QoL was generally maintained rather than substantially improved. Waterhouse et al. [30] and Makharadze et al. [34] reported that patients receiving targeted therapy, immunotherapy, or chemo-immunotherapy combinations maintained stable QoL despite intensive treatment. Similarly, Fiteni et al. [35] observed variability in QoL outcomes depending on chemotherapy regimens, highlighting that treatment modality influences patient-reported outcomes. These findings suggest that modern systemic therapies, when combined with supportive care, may help prevent deterioration in QoL.

Multimodal interventions combining education, rehabilitation, and psychosocial support demonstrated synergistic effects. Granger et al. [27] and Gu et al. [36] reported improved postoperative recovery, reduced symptom burden, and enhanced functional outcomes following integrative rehabilitation programs. Additionally, Kenis et al. [37] showed that geriatric assessment combined with structured coaching significantly improved QoL in older cancer patients receiving systemic therapy. These findings support a holistic, multidisciplinary approach to lung cancer care.

The predominance of low-risk randomized controlled trials strengthens confidence in several reported benefits of educational and supportive interventions; however, moderate-risk observational and pilot studies require more cautious interpretation due to potential confounding and limited statistical power. Interpretation of findings should consider methodological quality, as randomized controlled trials with low risk of bias provided stronger and more reliable evidence of benefit compared to observational studies with moderate risk of bias, which may be more susceptible to confounding and reduced internal validity.

Despite the overall positive direction of findings, some inconsistencies were observed across studies. Not all interventions demonstrated significant improvements in all QoL domains, with certain studies reporting limited or non-significant changes in global health status and symptom-specific scores. In particular, variability was noted in fatigue and emotional functioning outcomes across similar intervention types, suggesting that these domains may be less responsive or influenced by baseline patient characteristics and disease stage. Additionally, differences in study design, intervention intensity, and follow-up duration contributed to variability in reported effects, even among studies evaluating comparable interventions such as exercise or educational programs. These discrepancies highlight the complexity of measuring QoL outcomes in heterogeneous lung cancer populations and should be considered when interpreting the overall findings. Overall, the findings support the integration of structured patient education, exercise, and psychosocial interventions into routine lung cancer care, despite variability in study design and patient populations.

Strengths

This review has several strengths. First, it focuses specifically on studies utilizing the validated EORTC QLQ-C30 and QLQ-LC13 instruments, allowing for greater consistency and comparability of QoL outcomes across studies. Second, although narrative in nature, a systematic search strategy was employed, enhancing the transparency and reproducibility of the study selection process. Third, the review integrates findings from multiple types of interventions, including patient education, exercise, and psychosocial support, providing a comprehensive overview of factors influencing QoL in lung cancer patients. Finally, the findings have clear clinical relevance, supporting the incorporation of structured educational interventions into routine oncological care.

Limitations

The present review has several limitations that should be considered when interpreting the findings. First, there was substantial variation in intervention characteristics, including the duration, intensity, and delivery of educational programs, which limits comparability across studies [14].

Second, outcome measurement was not fully standardized, as several studies either did not consistently apply the EORTC QLQ-C30 and QLQ-LC13 instruments or used different scoring approaches, thereby limiting comparability of QoL outcomes. Third, many included studies were non-randomized or had relatively small sample sizes, which reduces the robustness and generalizability of the evidence.

The risk of bias assessment indicated that while several randomized controlled trials demonstrated low risk of bias, a few observational and smaller studies were associated with moderate risk, primarily due to confounding and methodological limitations. In addition, the narrative design of this review precludes formal quantitative synthesis and limits the ability to draw causal inferences.

Furthermore, the inclusion of studies across diverse clinical settings-including postoperative recovery, systemic therapy, rehabilitation, and advanced disease-introduces significant clinical heterogeneity. While this enhances the applicability of findings across the lung cancer care continuum, it also limits direct comparison between intervention types and patient subgroups.

Inconsistent reporting of QLQ-C30 and QLQ-LC13 outcomes across studies limited the ability to compare effect sizes and precluded meta-analytic synthesis. Finally, the findings may be affected by publication bias, as studies reporting positive effects of educational and supportive interventions are more likely to be published, potentially leading to an overestimation of their true impact.

## Conclusions

Patient education and supportive interventions appear to have a beneficial impact on health-related QoL in patients with lung cancer. The available evidence suggests that structured educational, psychosocial, and rehabilitation-based interventions are associated with improvements in symptom control, emotional functioning, and overall QoL across different stages of disease and treatment. These findings support the integration of patient-centered supportive care as an essential component of multidisciplinary lung cancer management.

From a clinical perspective, early incorporation of standardized patient education, focusing on disease understanding, symptom self-management, and treatment adherence, alongside psychosocial and physical rehabilitation strategies, may enhance patient outcomes and functional recovery, particularly when initiated at diagnosis or at treatment initiation. However, implementation should consider heterogeneity in intervention design, patient populations, and outcome reporting. Importantly, supportive and educational interventions should be tailored according to treatment modality, disease stage, and individual patient needs, as postoperative, systemic therapy, and advanced disease settings present distinct physical and psychosocial challenges. Further large-scale, well-designed randomized controlled trials with standardized outcome reporting are needed to better define the magnitude, consistency, and durability of these benefits.
